# Electrokinetic Chromatography‐based Micro Methods for Separation and Physiochemical Characterization of Very Hydrophobic Pharmaceuticals

**DOI:** 10.1002/jssc.70127

**Published:** 2025-04-18

**Authors:** Robert Minkner, Hermann Wätzig

**Affiliations:** ^1^ Institute of Medicinal and Pharmaceutical Chemistry TU Braunschweig Braunschweig Germany; ^2^ Haupt Pharma Wülfing Member of the Aenova Group Gronau Germany

**Keywords:** electrokinetic chromatography, hydrophobicity, MEEKC, pharmaceuticals

## Abstract

This study established an efficient separation of the highly hydrophobic analytes fenofibrate, orlistat, lumefantrine and a micelle marker using microemulsion electrokinetic capillary chromatography. A starting buffer with sodium dodecyl sulphate, heptane and butan‐1‐ol resulted in a partial separation of the analytes. Various experimental parameters were investigated, including voltage, sample loading time, buffer composition and temperature. An excellent separation was achieved, and compounds with hydrophobicity of up to a calculated log *P* of 10.2 (est. 12) could be clearly distinguished. Due to observed variabilities in retention time when changing the capillaries, it is advisable to calibrate with a standard mixture before each measurement series.

**Abbreviations**
BFS: bare fused silica
CE: capillary electrophoresis
CMC: critical micellar concentration
CTAB: cetrimonium bromide
CV: coefficient of variation
DAD: diode array detector
DoE: design of experiments
EOF: electroosmotic flow
HPLC: high‐performance liquid chromatography
MEKC: micellar electrokinetic chromatography
MEEKC: microemulsion electrokinetic chromatography
SDS: sodium dodecyl sulphate

## 1 Introduction

### 1.1 Hydrophobicity Determination

Hydrophobicity refers to the tendency of a molecule or moiety to dissolve in a lipophilic environment. Hydrophobicity plays an essential role in the liberation, absorption, distribution, metabolism and excretion processes of chemical compounds in biological systems, making it one of the most important physicochemical properties to determine during drug discovery and development [1]. Hydrophobicity is usually measured by evaluating the partitioning behaviour in a biphasic system, such as *n*‐octanol/water (o/w). Various hydrophobicity indices can be obtained, depending on the biphasic system used, with the o/w partition coefficient (log *P*_o/w_, also known as log *K*_o/w_ or log *P*) being the most commonly used. Whereas logP is only valid for the neutral form of the analyte, while the distribution coefficient log *D* is pH dependent and refers to an ionisable analyte present at a specific pH [1, 2, 3]. Where clog*P* stands for a calculated log *P* value and depends on the algorithms used, which may differ. For example, xlog*P* algorithms are often used. clog*P* is frequently used when no method is available for a proper experimental log *P* determination.

Hydrophobicity is also a critical parameter for the assessment of chemical safety, and two common methods for its measurement are direct measurement using shake‐flask techniques and the correlation approach using high‐performance liquid chromatography (HPLC) [1]. However, other experimental methods may be used provided they have an acceptable level of analytical quality. Many chromatographic systems have already been used to investigate hydrophobicity. These systems require calibration with reference substances, which function as standards [1, 2, 3]. However, the precision of estimating log *P*_o/w_ values is significantly impacted by the similarity between calibration standards and sample compounds. Unlike HPLC techniques, micellar electrokinetic chromatography (MEKC) and microemulsion electrokinetic chromatography (MEEKC) measurements enable precise correlation with log *P*_o/w_ without the requirement of supplementary molecular descriptors [4], and a simple calibration with (best suited) standards seems sufficient [2, 3, 5]. Several publications have shown that these are excellent indirect methods for determining the logP for pharmaceutical drugs/analytes [1, 2, 3, 4, 5, 6, 7].

### 1.2 Micellar Electrokinetic Chromatography

MEKC is a special capillary electrophoresis (CE) method in which separation occurs in an electrolyte solution containing a surface‐active substance at a concentration above the critical micellar concentration (CMC), which was first introduced by Terabe et al. in 1984 [8] and then fully published in 1985 [9]. The molecules of the solute are distributed according to their partition coefficients between the aqueous buffer solution and the pseudo‐stationary phase of micelles. Therefore, this technique can be considered as a combination of electrophoresis and chromatography [5, 10]. It can be used for the separation of both neutral and charged solutes while maintaining the separation performance, speed, and instrumental precision that characterise capillary electrophoresis [5, 10–12]. MEKC not only provides a versatile separation mode, but also a simple way to work with poorly soluble analytes, as is the case of Sudan dyes [13], alkaloids [14], or some pesticides such as bioallethrin or hexaconazole [15]. The use of MEKC has been extensively discussed and reviewed in the literature, also sometimes mentioning log *p*‐value estimations. A recently published review of method development [12] and the numerous reported applications in the literature are recommended [16, 17, 18, 19, 20, 21, 22, 23, 24, 25, 26].

### 1.3 Microemulsion Electrokinetic Chromatography

The same principle is used in MEEKC, except that instead of micelles, microemulsion droplets form the pseudo‐stationary phase, which was introduced by Watari in 1991 [27]. Compared to MEKC, which has an almost fixed and uncontrollable separation window due to the absence of an inner oil core that allows structural flexibility, the MEEKC elution range can be more easily modified for example, by adjusting the surfactant concentration [5, 10, 11, 28, 29]. In addition, MEEKC offers the unique advantage of increased hydrophobic volume/surface area as a result of the presence of an oil core, which enhances the solubilisation capacity and separation of various hydrophobic substances. Furthermore, the oil phase in the microemulsion structure can improve fluidity, resulting in an increase in the mass transfer rate and a higher separation efficiency [10, 11, 12, 28, 30]. It has been found that MEEKC can have a better correlation with the o/w coefficient than MEKC [4]. And within this, sodium dodecyl sulphate (SDS) correlates better with this coefficient than cetrimonium bromide (CTAB) [4]. When SDS, *n*‐butanol and *n*‐heptane were then combined to form the microemulsion phase, the best agreement was achieved with the octanol/water partition coefficient, at least in some cases [2]. However, some of these advantages of MEEKC over MEKC have yet to be confidently confirmed [11, 30]. It is generally assumed that MEEKC is better than MEKC for separating hydrophobic analytes.

Since MEEKC was introduced, most of the MEEKC methods are still based on this initial setup [31], and the rationale behind the microemulsion is still not fully understood, even though more and more progress is being made [5, 11, 29, 30]. For a detailed overview, also with regard to the structure of microemulsions, we refer to Huie [11]. The composition of water and oil determines the structure of the microemulsions, which can be either oil‐in‐water or water‐in‐oil, depending on the ratio of the two substances. It is important to note that the microstructures of microemulsions remain highly variable, with the water and oil domains rapidly changing in size and shape, while the amphiphilic molecules also rapidly change their location within the microemulsion systems [11]. Microemulsions usually consist of oils dispersed in aqueous solutions stabilised by surfactants. Compared to the micelles, the inner oil core is larger and they can be more modified. This usually results in higher specificity and separation efficiency, but on the downside, microemulsions are less stable and have a tendency to de‐emulsify [11, 32]. It is also possible to partially modify micelles with the addition of organic solvents. This type of modified micelle is known as a solvent‐modified micelle or swollen micelle and can have a performance similar to that of a microemulsion [30, 32]. A MEEKC performance experiment using the design of experiments (DoE) showed that SDS and propan‐2‐ol as modifiers had the most significant effect on selectivity [33]. Similarly, another study investigated several microemulsion parameters, and their effect on hydrophobicity determination [6]. This result is interesting because none of the changes significantly affected the properties of the microemulsion or the similarity of the MEEKC systems to the classical logP_O/W_ distribution [2, 6].

MEEKC is the best method for determining hydrophobicity because it can analyse very small sample volumes. Injecting only nanolitres (maximal microlitres) in CE is feasible, making it ideal when working with limited amounts of substance, which is a common challenge faced by many (pharmaceutic) researchers [12]. By contrast, traditional methods like octanol/water partitioning or HPLC require significantly larger sample volumes, typically at least a full sample loop plus template, which can be prohibitive when dealing with scarce compounds. MEEKC's key advantage is that it allows for efficient utilization of valuable samples. Furthermore, MEEKC offers as mentioned several other benefits, including short analysis times, reduced solvent consumption, and the versatility to separate a wide range of compounds, making it an attractive technique for this study [12].

## 2 Materials and Methods

### 2.1 MEKC and MEEKC Buffers

Information on the composition of the MEEKC buffers, the reasons for their selection and their performance can be found in the corresponding (Supplementary) results section. The compositions of one MEEKC buffer are given as examples.

The buffer consisted of 7.69 mmol/L NaPO_4_, 115 mmol/L SDS, 0.91% [v/v] *n*‐Heptane, 23.74% [v/v] Butan‐1‐ol, 20.35% Acetonitrile, 55% [v/v] H_2_O, pH* 8.49: 0.05999 g NaH_2_PO_4_ × 2 H_2_O, 1.658185 g SDS, 0.455 mL *n*‐heptane, 11.88 mL Butan‐1‐ol, 10.175 mL Acetonitrile, 27.5 mL ultrapure water; afterwards the apparent pH* was manually adjusted to 8.5 (“DoE setup 5”). The pH* of the prepared buffers was adjusted manually with 1 mol/L NaOH, 0.5 mol/L NaOH or 2 mol/L HCl.

### 2.2 Pharmaceutical Active Compounds

The pharmaceutical compounds used for hydrophobicity determination were fenofibrate (Sigma‐Aldrich, Steinheim, Germany), lumefantrine (Acros Organics, Geel, Belgium), and orlistat (Formosa Laboratories Inc., Taoyuan, Taiwan). For the characteristics of all analytes and markers see Table 1.

**TABLE 1:** (Calculated) log *P* values and detection wavelengths for analytes and markers based on literature; (c)log*P* = (calculated) log *P* value and the calculated log *P* value depends on the algorithm/software used (e.g. xlog*P*3‐AA or ALOGPS); Information is mainly from the online databases Drugbank ([34]) and Pubchem ([35]), especially for log *P* values, and from some publications.

Classification	Substance	(c)log*P*	Wavelengths for detection (nm) from literature	Reference
Analytes	Fenofibrate	5.3 (xlog*P*3‐AA) 4.86 (ALOGPS)	280 and 290 nm	[34, 35, 43, 44]
	Orlistat	10 (xlog*P*3‐AA) 7.61 (ALOGPS)	212 nm; 450 and 205 nm	[34, 35, 45, 46]
	Lumefantrine	8.7 (xlog*P*3‐AA) 8.34 (ALOGPS) (pH↑→log *P*↑)	290 and 316 nm; 234 nm	[34, 35, 47]
EOF marker	Dimethylformamide (DMF)	−1 (xlog*P*3‐AA) −0.77 (ALOGPS)	(In literature: 197.4 nm) 195 nm	[34, 35]
	Thiourea	−0.8 (xlog*P*3‐AA)	(In literature: 241 nm) 195 and 234 nm	[35]
Micelle/ Microemulsion marker	Quinine (hydrochloride)	2.9 (xlog*P*3‐AA) 2.82 (ALOGPS) 2.6/3.44 (experimental)	(In literature: 205, 232, 277, 321 and 332 nm) 220 and 274 nm	[34, 35, 48]
	DL‐Alpha‐Tocopherol (Vitamin E)	10.7 (xlog*P*3‐AA) 12.2 (est.)	(In literature: 294 nm) 205 and 290 nm	[35]
	Menaquinone 4 (K2)	8.9 (xlog*P*3‐AA)	(Also in literature: 195, 266, 267 and 278 nm) 195 and 205 nm	[35]
	Diisodecyl phthalate	10.6 (xlog*P*3‐AA) 10.36 (est.)	(In literature: 275 nm with tailing beyond 290 nm) 195, 205 and 290 nm	[35]

### 2.3 Micelle Markers

As micelle markers, Diisodecyl phthalate (Sigma Aldrich, München, Germany), Menaquinone 4 (Vitamin K2 derivate; Sigma Aldrich, München, Germany) and Quinine hydrochloride dihydrate for synthesis (MERCK‐Schuchardt, Hohenbrunn, Germany) were used.

### 2.4 Electroosmotic Flow Markers

As an electroosmotic flow (EOF) marker, Thiourea 99+ for analysis (Acros Organics, Geel, Belgium) was used.

### 2.5 Other Reagents and Materials

Other reagents used included: Acetone HPLC grade (Fisher Chemical, Schwerte, Germany), Acetonitrile LC‐MS grade (VWR, Darmstadt, Germany), Butan‐1‐ol (Thermo Scientific, Kandel, Germany), Cetyltrimethylammonium bromide (“CTAB”; synonym: Hexadecyltrimethylammonium bromide; Thermo Scientific, Kandel, Germany), Dodecylsulfate‐sodium‐salt electrophoresis grade (SDS; SERVA, Heidelberg, Germany), Ethanol absolute (Sigma Aldrich, München, Germany), *n*‐Heptane HPLC grade (99+%) (Thermo Scientific, Kandel, Germany), Propan‐2‐ol ≥99.5% (Fisher Chemical, Schwerte, Germany) and Sodium Hydroxide (Sigma Aldrich, München, Germany).

### 2.6 Ultrapure Water

Drinking water was treated with Geno MSR‐tronic (Grünbeck Wasseraufbereitung GmbH, Höchstädt, Germany), a reverse osmosis system, to produce demineralised water. It was then processed into ultrapure water using the Arium pro VF (Sartorius, Göttingen, Germany).

### 2.7 Separation Capillaries

For the individual capillary lengths, please see the corresponding figure legends in the results sections. Bare fused silica capillaries TSP050375 3, 363‐10 Fused Silica (Polymicro Technologies, Phoenix, Arizona, USA) in lengths ranging from 41.5 cm (effective length: 31.5 cm) to 43.2 cm (effective length: 33.2 cm) were used. Three different batches of this type of capillary were used, as mentioned in the manuscript. The TSP025375 Fused Silica capillaries (Polymicro Technologies, Phoenix, Arizona, USA) had a length of 40.9 cm (effective length: 30.9 cm), and the TSP075375 3, 363‐10 Fused Silica (Polymicro Technologies, Phoenix, Arizona, USA) had a length of 43.2 cm (effective length: 33.2 cm).

### 2.8 Other Equipment

Other used equipment were: Microcentrifuge VWR Micro Star 12 (VWR International bvba, Leuven, Belgium), Sartorius Analytical Lab Scale Digital Balance BP211D (Sartorius Lab instruments, Göttingen, Germany), Sartorius analytics Typ A12OS‐D1 balance (Sartorius Lab Instruments, Göttingen, Germany) and Sartorius MC5 microbalance (Sartorius Lab instruments, Göttingen, Germany).

### 2.9 Software

The Clarity Chromatography software from version 8.6.1.49–8.6.1.69 (DataApex, Prague, Czech Republic), Microsoft Office Professional Plus 2019 (Microsoft Cooperation, Redmond, WA, USA), Microsoft Office Student 2021 (Microsoft Cooperation, Redmond, WA, USA) and Minitab 19.2020.1 (64‐bit) (Minitab, State College, PA, USA) were used.

### 2.10 Databases

Drugbank online (https://go.drugbank.com/) [34] and the Pubchem database (https://pubchem.ncbi.nlm.nih.gov/) [35] were used for substance information.

### 2.11 MEKC and MEEKC

For the experiments, the Prince CE Next 870 (Prince Technologies, Emmen, Netherlands) was used. The samples for injection were weighed together into an Eppendorf microtube and then dissolved in 1 mL of the mobile phase. Additionally, the dissolution process was supported by ultrasonication in a water bath. Afterwards, the sample solution was then centrifuged with 12300 ×g for 2 min (Microcentrifuge VWR Micro Star 12; VWR international bvba, Leuven, Belgium). The supernatant was injected.

All CE methods were performed at 22°C unless otherwise stated. To condition the capillary before a measurement series, 1 mol/L NaOH was rinsed at 1500 mbar for 1800 s. This was followed by ultrapure water at 1500 mbar for 900 s. This was continued by a mobile phase flush at 1500 mbar for 900 s. Finally, a blank run was performed using the respective mobile phase at 15 kV for 1800 s. Subsequently, the first step was changed to 1 mol/L NaOH at 1250 mbar for 2400 s and then up to 3600 s.

The cleaning method was performed with 1 mol/L NaOH at 1500 mbar for 1800 s. This was followed by rinsing with ultrapure water at 1500 mbar for 1200 s. The capillary ends were then immersed in ultrapure water.

The applied voltage and running time varied and are mentioned in the text or figure legends. However, in general, the mobile phase was flushed at 1500 mbar for 240 s, and then the sample was injected at 50 mbar for 6 s. This was followed by a short injection of the mobile phase at 50 mbar for 2 s. The capillary end was then dipped into the mobile phase inlet and outlet vials and the run was performed. During development, the time for the first rinse of the capillary with the mobile phase was increased to 420 s.

Detection was performed using a diode array detector (DAD) with a deuterium lamp. The wavelength used is given in the text and figures and had a bandwidth of 1 nm. The scan type was absorbance mode and integration was performed in 80 milliseconds with 4 scans per period and a frequency of 10 Hz. The full spectrum ranged from 190 to 610 nm. The offset of the spectra was mainly in the range of 1800–2200.

## 3 Results and Discussion

### 3.1 Focusing on MEEKC

First, several attempts were performed with MEKC setups as a starting point, and then further developing to early MEEKC setups (See Sections S3a,b, including Figure S1) [36]. The rationale behind this approach is that the initial micellar environment was found to be hydrophilic and inadequate. This resulted in not only non‐solved compounds but also inadequate separation of the compounds. Consequently, the approach was switched to a MEEKC method. As the MEEKC approach was promising but not yet sufficient, a new type of MEEKC mobile phase was made based on the publication by Yin et al. [37], which allowed them to separate fat‐soluble vitamins. A borax‐based mobile phase was published, but here a phosphate‐based mobile phase was used, consisting of 7.69 mmol/L NaH_2_PO_4_, 42 mmol/L SDS, 0.8% (v/v) *n*‐heptane, 21% (v/v) butan‐1‐ol, 18% (v/v) acetonitrile and 60.2% (v/v) water at pH* 8.5. The SDS concentration in the publication was given as 1.2 g in 100 mL, which is calculated as 42 mmol/L [37].

The first results of this series were disappointing, as the EOF marker (thiourea) and quinine hydrochloride were multiple peak complexes, although separation of orlistat and lumefantrine was achieved (Figure S2A). It was assumed that the SDS concentration was not sufficient to stabilise the microemulsion and therefore, the peaks also did not have a Gaussian shape, contrary to the publication [37]. Thus, two new similar mobile phases were prepared, one with an increased SDS concentration of 70 mmol/L and the other with a change to 75 mmol/L cationic CTAB.

The 70 mmol/L SDS mobile phase was able to separate orlistat and lumefantrine. The 75 mmol/L CTAB buffer could also separate orlistat and lumefantrine, but the peak shape was worse and the retention time was longer (See Section **S**3c for details). Despite the acceptable results of the CTAB‐based mobile phase, it was not investigated further because the results were not as satisfactory as those of the SDS‐based mobile phase, which appeared to be easier and quicker optimised.

**FIGURE 1:**
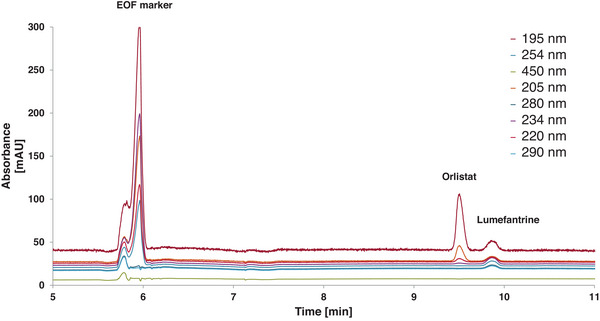
Improved microemulsion electrokinetic chromatography (MEEKC) separation with only 52% (v/v) water. The chromatogram shows only the relevant part. The capillary length was 43.1 cm (effective length: 33.1 cm). The sample was dissolved in the mobile phase and loaded with 50 mbar for 6 s. The running condition was 20 kV for 20 min. The MEEKC mobile phase consisted of 7.69 mmol/L NaH_2_PO_4_, 70 mmol/L sodium dodecyl sulphate (SDS), 0.97% (v/v) *n*‐heptane, 25.33% (v/v) butan‐1‐ol, 21.71% (v/v) acetonitrile, 52% (v/v) water, pH* 8.52. The sample consisted of 1.17 mg thiourea + 1.86 mg orlistat + 0.66 mg lumefantrine + 1 mL buffer.

However, repeatability problems arose as retention time shifts and unexplained baseline elevations were still major challenges. Therefore, the water content was reduced to obtain an even more hydrophobic mobile phase. The rationale for how the water/organic phase ratio was adjusted, can be seen in Section S3.1. Separation of orlistat and lumefantrine was obtained with a freshly prepared sample (Figure 1). However, the EOF marker had an undesirable fronting.

**FIGURE 2:**
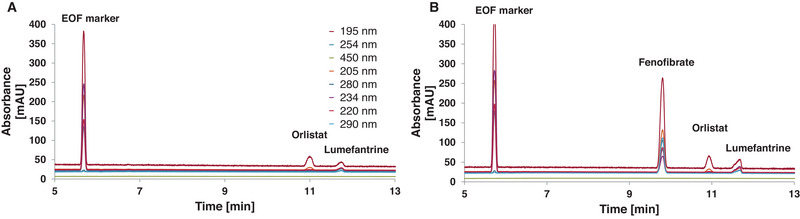
Improved microemulsion electrokinetic chromatography (MEEKC) separation with 60.2% (v/v) water but higher sodium dodecyl sulphate (SDS) concentration. The chromatograms show only the relevant part. For clarity, the legends in (B) are the same as in (A). Two chromatograms are shown, to demonstrate that repeatability can be achieved even with slightly different sample compositions. The substances are assigned to the peaks within the chromatograms. The capillary length was 43.1 cm (effective length: 33.1 cm). The sample was dissolved in the mobile phase and loaded at 50 mbar for 6 s. The MEEKC mobile phase consisted of 7.69 mmol/L NaH_2_PO_4_, 85 mmol/L SDS, 0.8% (v/v) *n*‐heptane, 21% (v/v) butan‐1‐ol, 18% (v/v) acetonitrile, 60.2% (v/v) water, pH* 8.46. In (A) the running condition was 20 kV for 30 min and the sample consisted of 1.65 mg thiourea + 0.95 mg orlistat + 0.78 mg lumefantrine + 1 mL buffer. In (B) the running condition was 20 kV for 20 min and the sample consisted of 2.41 mg thiourea + 1.02 mg fenofibrate + 1.05 mg orlistat + 1.04 mg lumefantrine + 1 mL buffer.

The fronting of the EOF marker thiourea was assumed due to the lower water ratio, as the EOF marker is less hydrophobic. Therefore, a new mobile phase containing 3% more water was used, but this did not improve the fronting of the EOF marker but still achieved the separation of orlistat and lumefantrine. It was hypothesised that the instability of the microemulsion was due to an insufficient amount of surfactants as the low ratio of water demands more SDS. Therefore, a MEEKC mobile phase was prepared with a slightly higher SDS concentration (+15 mmol/L), but not excessively high, as this would have a detrimental effect on the EOF due to the negative charge. The new mobile phase consisted of 7.69 mmol/L NaH_2_PO_4_, 85 mmol/L SDS, 0.8% (v/v) *n*‐heptane, 21% (v/v) butan‐1‐ol, 18% (v/v) acetonitrile and 60.2% (v/v) water at pH* 8.46. This mobile phase improved the solubility of lumefantrine. A very good separation of fenofibrate, orlistat and lumefantrine was achieved (Figure 2).

Next diisodecyl phthalate (xlog*P*3 = 10.6) and menaquinone 4 (vitamin K2 derivative; xlog*P*3 = 8.9) were investigated as micelle markers to see if the new method is able to separate them from lumefantrine. Diisodecyl phthalate gave a strong signal with a small tailing, but menaquinone 4 however, was preferable, as the signal was also strong with a “perfect” Gaussian shape (**data not shown**), but both were usable. However, the separation from lumefantrine was incomplete, and the analysed run data showed that the repeatability of the retention time and especially the retention time difference of the EOF and the micelle marker were still too inconsistent (Figure S3).

### 3.2 DoE to Optimize the MEEKC Mobile Phase

From these results, the shown data indicated that reducing the water content and increasing the SDS concentration might be the best options to further improve the method. Therefore, a DoE was carried out to determine whether the identified factors, water ratio, and SDS concentration sufficiently affected the separation efficiency. The water ratio levels were set at 55 and 45% with a central point of 50%, and the SDS concentrations were set at 85 and 115 mmol/L with a central point of 100 mmol/L (See Table S1). The pH* was set to 8.5. A full factorial design with two factors was used, and each run was replicated in triplicate. The collected and partially manually numbered results are listed in Section S3.2a.

In Section S3.2b, the results are described in detail. In short, three DoE setups had a successful separation. DoE setup 3 with 100 mmol/L SDS and 50% water as the centre point was able to separate lumefantrine from the micelle marker in 25 min with a stable baseline. However, the EOF marker had a double peak (**data not shown**). DoE setup 4 with 85 mmol/L and 55% water seems to have separated lumefantrine from the micellar marker in about 20 min (Figure S4A). The results of DoE setup 5 with 115 mmol/L SDS and 55% water were judged to be the best (Figure S4B). The disadvantage of the latter was that the separation took 32 min, but the advantage was that the retention time distance between lumefantrine and the micelle marker was the largest. However, it should be noted that in this series, the separation retention time decreased from around 32–29 min. DoE setup 5 had the highest resolution between lumefantrine and the micelle marker with 5.74 (SD = 0.1994), and the second best had DoE setup 3 with 3.06 (SD = 0.1363). The analysis time can be considered good, as in another study, the MEKC buffer had a separation time of 35 or 50 min, depending on whether the SDS had a concentration of 50 or 60 mmol/L, and the MEKC buffer had a water ratio of 90% (v/v) [38]. In comparison, the DoE setup 5 mobile phase used here with 115 mmol/L SDS and 55% (v/v) water could achieve a separation time of less than 20 min, depending on the capillary used, as shown in the next section, 2^nd^ passage.

### 3.3 Investigations to Improve Repeatability of Retention Time

**FIGURE 3:**
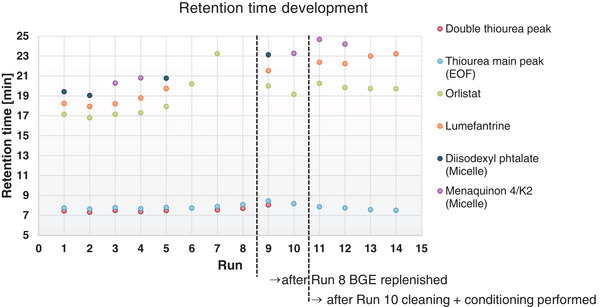
Trend chart of Retention times demonstrating the effect of interventions. The figure visualizes data of the DoE5 sequence (pH = 7.98). As described in the text, due to the weakened electroosmotic flow (EOF), some of the analytes could not be detected in the time available in some of the runs.

Repeatability was still a major task, as briefly described in Section S3.3a. Based on these experimentations, a repetition experiment study of the DoE setup 5 variant with a pH* of 7.98 surprisingly led to a double peak of the EOF marker (**partly shown i**n Figure 3) and the EOF strength was lost after five runs. After replenishing the inlet and outlet buffers after the eighth run, the EOF strength could only be partially recovered (**partly shown in** Figure 3). After an additional cleaning and conditioning step, the EOF could also not be completely recovered, but additionally, the signal intensity of the 195 nm wavelength increased considerably (**partly shown in** Figure 3), and the only difference was that the two micelle markers were used alternating in this series. The double peak of the EOF marker simply disappeared without any cognisable relation to the interventions. Retention times were plotted on a graph to visualise the trend with actions taken to recover the EOF (Figure 3). The comparison of the pH investigations, at pH* 9.0, 8.5 and 8.0 (**data not shown**), showed that a pH* of 8.5 is more advantageous in terms of reproducibility, as up to five runs were possible without replacing the mobile phase.

**FIGURE 4:**
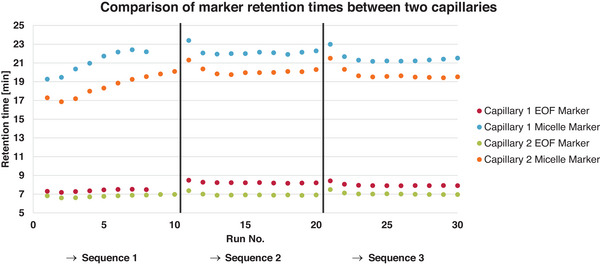
Comparison of marker retention times between two capillaries. Three sequences were performed on two different capillaries cut in series from the same batch of capillaries. One sequence consisted of conditioning, 10 runs with the same sample and different inlet and outlet vials each time, and finally cleaning. Note that for sequence 1 on the first capillary, only eight runs were performed due to the lack of a mobile phase. The electroosmotic flow (EOF) marker was thiourea and the micellar marker was diisodecyl phthalate. The vertical black bars indicate the end of the sequence.

The final attempt (after Section S3.3b) mentioned in this study to increase repeatability was to replenish the inlet and outlet vials after each run. This was done to eliminate the possibility of buffer compromise due to the change in the ionic strength. Then, two capillaries were prepared and cut in series directly from the same capillary batch. During the initial 10 consecutive runs with conditioning, cleaning, and vial changes for the first capillary, the previously prepared DoE setup 5 buffer only supported eight additional inlet/outlet pairs. Due to frequent DAD detector offset adjustments to manage high signal intensity at 195 nm, retention times were determined using the 254 nm wavelength to minimize data interpretation bias. The results of this experiment can be seen in Figure 4. The chromatograms look similar to Figure 2 (and Figure S4) but with faster retention times. For both used capillaries, the retention time becomes stable after five or six runs. For capillary 1 the plateau was reached after about the fifth run with *t* ≈ 22.16 min for the micelle marker and for capillary 2 the plateau was reached after about the sixth run with *t* ≈ 19.24 min. It is also interesting to note that at the beginning of the second sequence, the retention time of the EOF marker slightly increased compared to the first sequence. Otherwise, as already known, the micelle marker is more affected by the retention time variance than the EOF marker.

If all runs of capillary 1 were evaluated, the average retention time for the EOF marker was 7.90 min (SD = 0.3660), for the micelle marker, 21.62 min (SD = 0.8740) and the average retention time difference was 13.73 min, which is the usable separation window. However, if the first five runs were excluded to assume equilibration, the average retention time for the EOF marker was 8.02 min (SD = 0.2648), for the micelle marker 21.90 min (SD = 0.5643) and the average retention time difference was 13.88 min. Similarly, evaluating all runs of capillary 2, the average retention time for the EOF marker was 6.93 min (SD = 0.1740), for the micelle marker 19.49 min (SD = 1.0456) and the average retention time difference was 12.56 min. When the first six runs were excluded, the average retention time for the EOF marker was 6.99 min (SD = 0.1455), for the micelle marker, 19.93 min (SD = 0.5375) and the average retention time difference was 12.94 min. Apparently, a new capillary needs to be equilibrated with several runs before a stable retention time can be achieved.

Taken together, these data still show differences between capillaries which are most likely due to inhomogeneities as the capillaries were similar in length, even though the voltage was not adjusted and therefore the electric field was slightly different. However, the impact is only a minor theoretical possibility due to the aforementioned minor differences in length. This difference in retention time would affect the comparison of the calculated retention factor for different capillaries. However, calibrations with a standard set of compounds with a known log *P* value should allow a reliable determination of the hydrophobicity of substances, and once the capillary is equilibrated, it can be used for a long time.

## 4 Conclusions

A reproducible method was developed that was able to separate highly hydrophobic analytes from the markers. After several attempts using MEKC, MEEKC proved to be more successful. Based on the literature and after several step‐by‐step improvements, a suitable mobile phase was found. This was then further optimised using a DoE approach, achieving an excellent separation. In the majority of cases, MEEKC methods are used to separate hydrophobic compounds in the range of 0.5–5.5 log *P* [39]. The method is also very useful to estimate the hydrophobicity of compounds of very low amounts.

In the follow‐up to writing up this manuscript, three publications were found with the same aim of separating very hydrophobic compounds by ME(E)KC ([40, 41, 42]; see also Section S3.4). These three publications have methods/mobile phases that concentrate on separating very hydrophobic analytes, but it is not clear if they are also useful for hydrophobicity determinations.

It is important to note that there is still a remaining variability in the retention times, which is not yet fully understood. In general, these are partly due to capillary wall effects, mobile phase depletion (which we were able to exclude in this case), and necessary equilibration. The observed limited reproducibility has been reported for MEEKC before [4], and there is still not much reported about MEEKC in the literature. However, the latest mentioned experiments (section 3.3) have significantly stabilised the retention time and identified the main cause. Newly prepared capillaries require a few equilibration runs before the retention time is stable, depending on their individual properties. If not, the comparability of the retention times and in consequence, the retention factor will be affected.

To increase comparability between different capillaries, it should be possible to calibrate with substances of known log *P* so that the individual retention factors obtained are converted into calculated logP values for the analyte, which should then be comparable between different capillaries. The method developed can now be used to experimentally determine the hydrophobicity of these and other pharmaceutical compounds and compare them, if available, with the literature.

## Author Contributions

Robert Minkner performed the experiments, wrote the manuscript, and designed the study. Hermann Wätzig supervised the entire project and revised the manuscript. All authors read and approved the final manuscript.

## Conflicts of Interest

Despite Robert Minkners current association with Haupt Pharma Wülfing, a Member of the Aenova Group, all experiments were conducted at the Institute of Medicinal and Pharmaceutical Chemistry at the TU Braunschweig and have no relation to the current employer.

## Supporting information



Supporting Information

## Data Availability

All relevant data generated or analyzed during this study are included in this published article and its additional file. The missing datasets used and/or analyzed during the current study are available from the corresponding author upon reasonable request.
