# Supplementary material for: Electrokinetic Chromatography‐based Micro Methods for Separation and Physiochemical Characterization of Very Hydrophobic Pharmaceuticals
Source: J Sep Sci. 2025 Apr 18;48(4):e70127. doi: 10.1002/jssc.70127 (PMC12007571; doi:10.1002/jssc.70127)
Supplement: Supplementary file 1 — Supporting Information [file JSSC-48-e70127-s001.pdf]

Supplementary Material for: Electrokinetic chromatography-based micro methods for separation and physiochemical characterization of very hydrophobic pharmaceuticals

Robert Minkner[1,2], Hermann Wätzig[1,*]

[1] Institute of Medicinal and Pharmaceutical Chemistry, TU Braunschweig, Beethovenstr. 55, 38106 Braunschweig, Germany

[2] Haupt Pharma Wülfing, Member of the Aenova Group, 31028 Gronau (Leine), Lower Saxony, Germany

[*] **Corresponding author:** Telephone: +49-531 - 391 2764 (Prof. Hermann Wätzig).
E-mail address: h.waetzig@tu-bs.de (Prof. Hermann Wätzig)

Short introduction

In addition to the information in the main manuscript, we provide here additional information that we thought were interesting to mention in the main manuscript but which were too long to discuss in detail there. Here the aspects are listed in the same order as in the main manuscript.

3. Results and discussion

a. Pre-liminary MEKC experiments

To investigate the separation of the highly hydrophobic analytes fenofibrate, orlistat, and lumefantrine (for characteristics, see **Table 1**), MEKC methods were developed using bare fused silica capillaries with an inner diameter (I.D.) of 50 µm. Pre-liminary experiments lead to the starting point of the experiments in the following passage (**data not shown**). During this, Thiourea was established as EOF marker. No suitable micelle marker was found yet, so quinine hydrochloride was temporarily continually used (**data not shown**).

In the early experiments the analytes could not be properly suspended and separated in the mobile phase's, this led to the conclusion that the hydrophobicity of the mobile phase should be increased so that the analytes are better solubilised and interact less with the micelles. For this purpose, several simple mobile phases, with different organic modifier concentrations were investigated. Ethanol as organic modifier was investigated in the concentrations of 15, 25 and 35 % (v/v) ethanol. 2 % (v/v), 5 % (v/v), 8 % (v/v) and 12 % (v/v) butan-1-ol were followed mobile phases. As example for the detailed composition, the 12 % butanol buffer consisted of 12 % (v/v) Butan-1-ol, 30 mmol/l SDS, 7.69 mmol/l $NaPO_4$, pH* 8.5: 12 ml Butan-1-ol, 0.86514 g SDS, 0.11998 g $NaH_2PO_4 \times 2\ H_2O$, 88 ml ultrapure water; pH* manually adjusted to 8.5. However, none of these simple MEKC mobile phases could separate the analytes (**data not shown**). Interestingly, the required retention time decreased significantly with increasing

butan-1-ol concentration, which is in line with the literature (1). Some problems regarding the separation were encountered, partly mentioned in the following. First, the 12 % (v/v) butan-1-ol mobile phase with 30 mmol/l SDS was tested on a used capillary. This resulted in an even shorter retention time for the analytes, but a shoulder fronting of the thiourea (EOF marker) and a weaker signal of quinine hydrochloride, which was partly due to the reduced weighted sample (**Supplement Figure 1A**). Contrary to expectations, the repetition of a previously successful separation with 8 % (v/v) butan-1-ol mobile phase failed to separate the analytes in a freshly prepared capillary, but with a mobile phase with 12 % (v/v) was successful. However, both had improved chromatograms compared to an “overused” capillary (**Supplement Figure 1B+C**). In addition, it was found that 20 kV seemed to slightly improve the peak form of thiourea, the EOF marker. Therefore, 20 kV was used in most cases, although the running time increased compared to 25 kV.

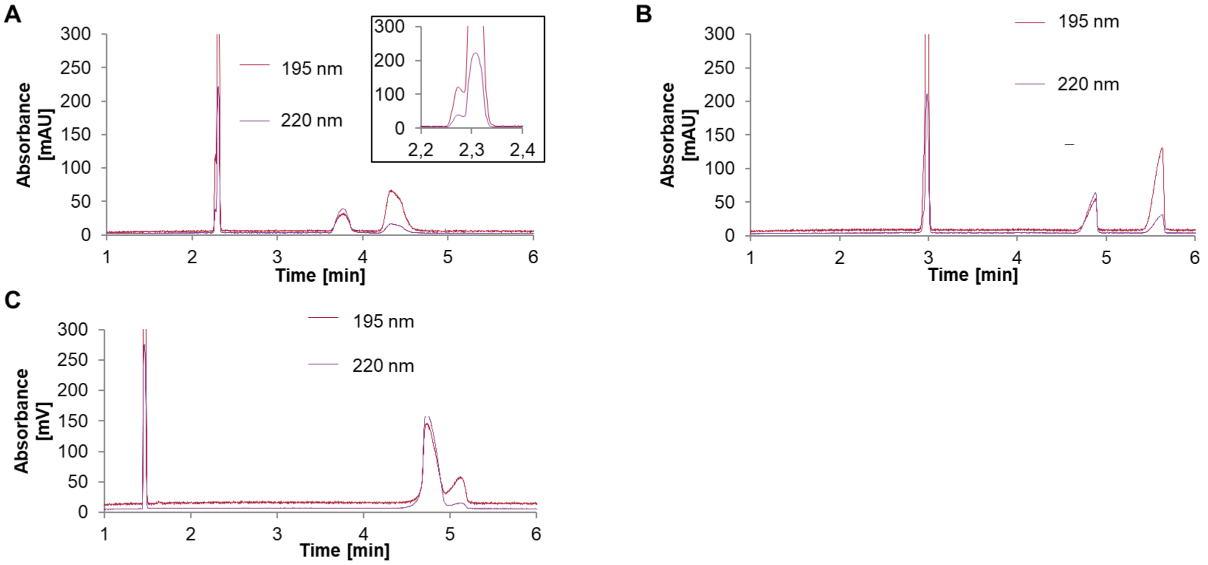

Supplement Figure 1 MEKC with different concentrations of butan-1-ol. The capillary length was 42.7 cm (effective length: 32.7 cm) for **A)** and 42.6 cm (effective length: 32.6 cm) for the others. The sample was dissolved in mobile phase and loaded with 50 mbar for 6 s. The running conditions were 25 kV for 15 min for **A)** + **C)** and 20 kV for 15 min for **B)**. For simplicity, only two wavelengths are shown in the chromatograms. In **A)** and **B)** the MEKC mobile phase consisted of 7.69 mmol/l NaH_2PO_4, 30 mmol/l SDS, 12 % (v/v) butan-1-ol, pH* 8.52. The sample consisted of 2.48 mg thiourea + 1.23 mg quinine hydrochloride + 1.38 mg fenofibrate + 1 ml buffer. In **A)** a box with the enlarged EOF marker thiourea is additionally shown. In **C)** the MEKC mobile phase consisted of 7.69 mmol/l NaH_2PO_4, 30 mmol/l SDS, 8 % (v/v) butan-1-ol, pH* 8.57. The sample consisted of 2.65 mg thiourea + 2.17 mg quinine hydrochloride + 1.18 mg fenofibrate + 1 ml buffer. As mentioned in the text, suddenly the separation of the analytes cannot be achieved and the peak shape is more gaussian at 20 kV than at 25 kV. Elution order was thiourea < quinine hydrochloride < fenofibrate.

Early experiments also showed that retention times were not stable and that there were shifts in retention time, suggesting that capillary effects on retention time were involved, with shifts possible in both directions, but especially to longer retention times (**data not shown**). The loss of the electrophoretic mobility also still happened when the mobile phase was optimized to strengthen the EOF.

To further improve the repeatability, optimisations for conditioning conditions were investigated, but with no relevant improvement. Therefore, since 40 minutes 1 mol/l NaOH conditioning was at least not detrimental, it was kept as standard (**data not shown**).

Suspecting capillary problems, further experiments with similar samples were carried out using different BFS capillaries from the same company but with inner diameters of 25 and 75 µm. However, no peaks other than thiourea were detected (**data not shown**). Runs with a 50 µm inner diameter BFS capillary also showed no abnormalities regarding voltage, power, inlet current or outlet current, but no analyte signals (**data not shown**). Therefore, it was decided to purchase a new batch of 50 µm BFS capillaries from the same company. This was done in the assumption that there would be batch differences and perhaps unexpected overstorage effects on the BFS capillary as the previous capillary batch allowed a better repeatability. Using a new batch of BFS capillaries (TSP050375 3, 363-10; 50 µm inner diameter) with a 20 mmol/l borate, 30 mmol/l SDS, 15 % (v/v) ethanol, pH* 8.51 buffer resulted in a well-functioning separation/EOF, but the separation results were not yet satisfactory (**data not shown**).

Other optimised MEKC buffers with Brij® 35, CTAB or methanol also did not improve the separation (**data not shown**).

b. First MEEKC experiments

The first investigated MEEKC mobile phase consisting of 7.69 mmol/l NaH_2PO_4, 75 mmol/l SDS, 0.8 % (v/v) n-heptane, 6.5 % (v/v) butan-1-ol, and 92.7 % (v/v) water at pH* 8.56. The 75 mmol/l SDS was based on Klotz *et al.* (2) 2.16 % (w/w) SDS, which is 1.08 g for 50 ml.

The MEEKC mobile phase was able to achieve some separation; although it was not clear if orlistat and lumefantrine were from each other separated and their supposed to be peak signals were quite weak, the latter most likely consisted of a double peak (**data not shown**). Interestingly, in these experiments, the retention time also accelerated with consecutive runs (**data not shown**). To address this further, 2 variants were created, in one 5 % (v/v) acetonitrile was added, and for the other, butan-1-ol was increased to 8 % (v/v). Lumefantrine was undetectable in chromatograms, indicating precipitation due to its hydrophobicity or potential co-elution with orlistat (**data not shown**).

c. MEEKC with SDS or CTAB

The mobile phase with 70 mmol/l SDS was able to perfectly separate orlistat and lumefantrine in approximately 15 min at 20 kV, and all peaks except quinine hydrochloride had an acceptable or even perfect Gaussian peak shape (**Supplement Figure 2B**). As the peak form of quinine hydrochloride was absolutely not gaussian shaped, indicating that it exists in multiple configurations and was not a suitable micellar marker, it was not included in any further samples. For the CTAB mobile phase, the general retention time was longer and quinine hydrochloride had a better peak shape, but the peak shape of orlistat was worse (**Supplement Figure 2C**). In addition, at t = 29–47 min three weak, long-stretched peaks were detected which could be associated with lumefantrine (**Supplement Figure 2D**). In both cases, interesting effects were observed in the sequence (without replenishing run buffer). In the case of the SDS mobile phase the retention time of the analytes increased with each run, but in the case of the CTAB mobile phase the retention time of the analytes decreased with each consecutive run (**Supplement Figure 2B + data not shown**). It is not clear whether these EOF changes are capillary surface related, similar to previous assumed problems, or due to the presence of analytes, surfactants, and/or other mobile phase compounds. Despite the usable results of the CTAB-based mobile

phase, it was not investigated further because the results were not as satisfactory as those of the SDS-based mobile phase method, which appeared to be easier and quicker to be optimised.

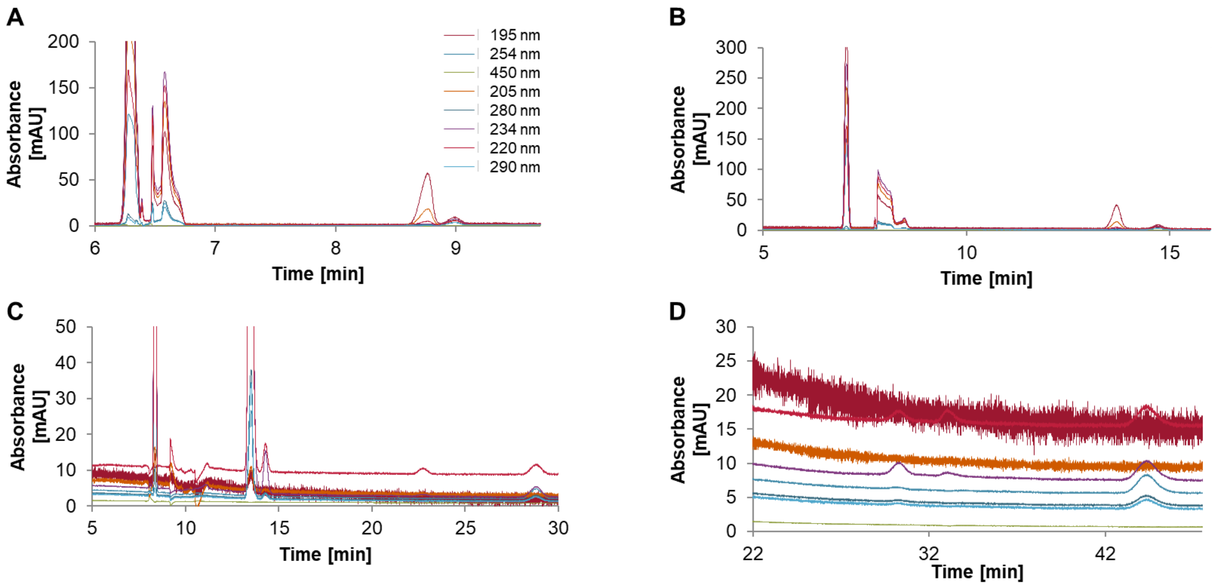

Supplement Figure 2 MEEKC optimisations based on the publication by Yin *et al*. Chromatograms show only the relevant part. For clarity, the legends are the same as in **A)**. The capillary length was 43.1 cm (effective length: 33.1 cm). The sample was dissolved in mobile phase and loaded with 50 mbar for 6 seconds. The running condition for **A)** and **B)** was 20 kV for 30 min. For **C)** it was -20 kV for 30 min and for **D)** -20 kV for 50 min. In **A)** the MEEKC mobile phase consisted of 7.69 mmol/l NaH_2PO_4, 42 mmol/l SDS, 0.8 % (v/v) n-heptane, 21 % (v/v) butan-1-ol, 18 % (v/v) acetonitrile, 60.2 % (v/v) water, pH* 8.50. The sample consisted of 1.89 mg thiourea + 2.27 mg quinine hydrochloride + 2.22 mg orlistat + 2.08 mg lumefantrine + 1 ml buffer. In **B)** the MEEKC mobile phase consisted of 7.69 mmol/l NaH_2PO_4, 70 mmol/l SDS, 0.8 % (v/v) n-heptane, 21 % (v/v) butan-1-ol, 18 % (v/v) acetonitrile, 60.2 % (v/v) water, pH* 8.49. The sample consisted of 2.25 mg thiourea + 2.39 mg quinine hydrochloride + 2.17 mg orlistat + 2.14 mg lumefantrine + 1 ml buffer. In **C)** + **D)** the MEEKC mobile phase consisted of 7.69 mmol/l NaH_2PO_4, 75 mmol/l CTAB, 0.8 % (v/v) n-heptane, 21 % (v/v) butan-1-ol, 18 % (v/v) acetonitrile, 60.2 % (v/v) water, pH* 8.5. The sample consists of 3.04 mg thiourea + 2.21 mg quinine hydrochloride + 1.95 mg orlistat + 1.86 mg lumefantrine + 1 ml buffer. Note that **D)** is a highly magnified version of a longer run which is the same as in **C)**.

3.1. Focusing on microemulsion electro kinetic chromatography – water/organic phase ratio adjustment

When the water content was reduced, the content of the corresponding inorganic components was increased in the same proportion. Here is an example of the calculation for the mobile phase with only 52 % water, decreasing down from 60.2 % water content. The difference in reduction from 60.2 to 52 % water is 8.2 %. The relative change in concentration of each component was calculated by dividing the change in water content (here 8.2 %) by the sum of all organic concentrations (here 39.8 %) and then multiplying this value by the original

concentration of the respective component. Therefore, 0.165 of 8.2 % corresponds to the original 0.8 % n-heptane amount, which, together in the new mobile phase, is 0.965 % (v/v) n-heptane. (Interestingly, another publication found 0.91% heptane to be the best, but it was a simpler MEEKC mobile phase (1).) Similarly, 4.327 corresponds to the 21 % butan-1-ol, which together make up 25.33 % (v/v). Finally, 3.709 corresponds to 18 % acetonitrile, which together make up 21.71 % (v/v). As a control calculation, 0.97, 25.33 and 21.71 % together with 52 % water give 100.01 %, which is sufficiently correct. The final mobile phase composition was 7.69 mmol/l NaH_2PO_4, 70 mmol/l SDS, 0.97 % (v/v) n-heptane, 25.33 % (v/v) butan-1-ol, 21.71 % (v/v) acetonitrile, 52 % (v/v) water, at pH* 8.52.

With reference to the main manuscript, it is briefly mentioned here that the separation of lumefantrine was still incomplete at this point and the analysed run data showed that the repeatability of the retention time and especially the retention time difference of the EOF and the micelle marker were still too inconsistent (**Supplement Figure 3**).

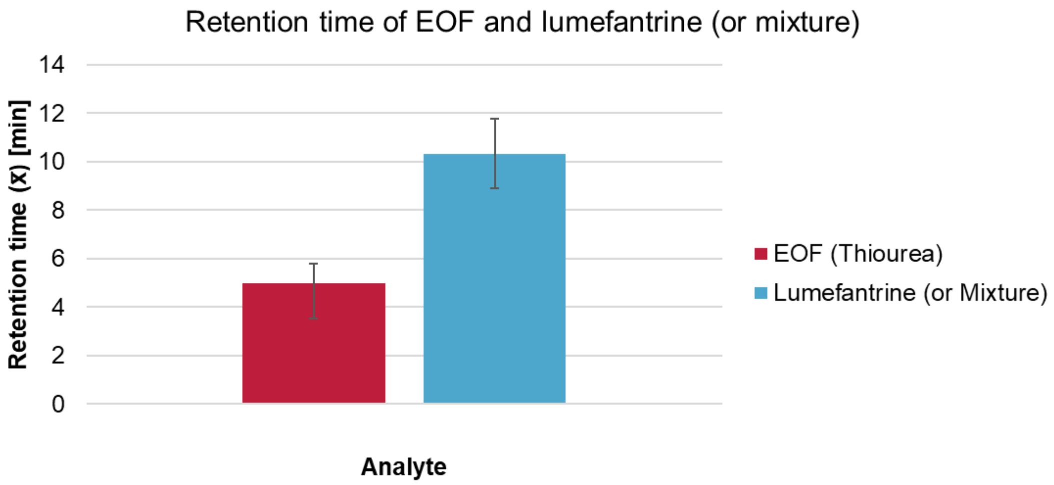

Supplement Figure 3 Comparison of retention time of EOF and lumefantrine (with/without in a mixture with a micelle marker). Based on two sequences with their EOF marker and lumefantrine or lumefantrine in a mixture with different micelle markers. Performed on two different days with a sequential injection of 2x3 or 2x2. Note that n = 10. The standard deviation is 0.8186 for EOF and 1.4240 for lumefantrine (mixture).

3.2. Design of Experiments

a. Design of Experiments

A full factorial design with two factors was used (**Supplement Table 1**), and each run was replicated in triplicate. The collected and partially manually numbered results are listed in **Supplement Table 2.** An additional comment on the classification of double peaks: As it was not easy or necessary to determine a correct value for the peak shape, a rough estimate was made, as only a sufficiently accurate determination would suffice if a peak was Gaussian enough to be useful. An artificial numbering system of 0-2 was used, with 0 being the best and 2 being the worst. A value up to 0.5 was still considered useful, but to minimise bias, the values were only given in intervals of 0.5.

Supplement Table 1 Overview of the DoE setup to optimize the MEEKC mobile phase.

Number	Random order	Water proportion	SDS concentration
**1**	1	45 %	85 mmol/l
**2**	3	45 %	115 mmol/l
**3**	5	50 %	100 mmol/l
**4**	2	55 %	85 mmol/l
**5**	4	55 %	115 mmol/l

Supplement Table 2 Collected results of the DoE setup to optimize the MEEKC mobile phase. The mobile phase was based on 7.69 mmol/l NaH_2PO_4 and a pH* of 8.5. The ratios of the different organic modifiers changed all in the same proportions. Explanation of the classification of double peaks: 0-2, where 0 is the best and 2 is the worst. A value up to 0.5 was still considered usable, but not more. As the classification was done manually and biased, it was only done in steps of 0.5 to avoid the bias as much as possible. Legend: *: No (useable) data could be obtained; Lum.: lumefantrine; Mic.: Micelle/micro emulsion droplets

Water (%)	SDS conc. (mmol/l)	t_{EOF} (min)	Double peak of EOF	$t_{Lum.}$ (min)	Double peak of Lum.	$t_{Mic.}$ (min)	Double peak of Mic.
**45**	115	13.172	1.0	*	*	31.652	2.0
**45**	115	*	*	*	*	*	*
**45**	85	13.172	2.0	*	*	22.440	2.0
**55**	85	7.842	0.5	16.932	0.5	18.170	0.5
**55**	115	8.017	0.5	27.197	0.0	31.077	0.0
**55**	115	7.853	0.5	26.075	0.0	29.755	0.0
**50**	100	9.235	1.0	21.727	0.5	23.523	0.0
**55**	115	7.763	0.5	25.357	0.0	28.818	0.0
**45**	85	13.218	2.0	*	*	23.928	2.0
**55**	85	7.750	0.5	16.392	0.5	17.887	0.5
**50**	100	9.370	1.0	21.967	0.5	23.732	0.0
**55**	85	7.733	0.5	16.318	0.5	17.690	.5
**45**	115	*	*	*	*	*	*
**45**	85	13.107	2.0	*	*	22.867	2.0
**50**	100	9005	1.0	20.832	0.5	22.465	0.0

b. Design of Experiments Results

The results are described in detail below, and the composition of the mobile phase is written down; however, to avoid confusion, they are numbered according to their normal order, as shown in **Supplement Table 1**.

DoE1 with 85 mmol/l SDS and 45 % water could not separate lumefantrine from the micellar marker, and it was also possible that the EOF marker developed a fronting. The baseline was hilly, and the actual peaks were even more so (**data not shown**). Similar results were obtained for DoE2 with 115 mmol/l SDS and 45 % water, and two of the three runs were automatically cancelled from the machine for safety reasons because the voltage was too high (**data not shown**). In contrast, DoE3 with 100 mmol/l and 50 % water as the centre point was able to separate lumefantrine from the micelle marker in 25 min with a stable baseline. However, the EOF marker had a double peak (**data not shown**). DoE4 with 85 mmol/l and 55 % water seems to have separated lumefantrine from the micellar marker in about 20 min. It could be that lumefantrine or the micellar marker developed a weak demerged peak, but they cannot be differentiated (**Supplement Figure 4A**). The results of DoE5 with 115 mmol/l SDS and 55 % water were judged to be the best as a separation was achieved (**Supplement Figure 4B**). The disadvantage was that the separation took during the DoE experiments 32 minutes, but the advantage was that the retention time distance between lumefantrine and the micelle marker was the largest. However, it should be noted that in this series, the retention time decreased from around 32 to 29 minutes. The resolution was calculated in relation to the *Ph. Eur.* between lumefantrine and the micelle marker. DoE5 had the highest resolution with 5.74 (SD = 0.1994), and the second-best separation had DoE3 with 3.06 (SD = 0.1363).

As mentioned, not all DoE setups were able to meet the desired responses, and some only partially, so the results table is incomplete (**Supplement Table 2**). This resulted in a non-orthogonal DoE design due to data loss, complicating interpretation. The lost data hindered

optimal DoE utilization, potentially confounding factor effects. While this limitation doesn't invalidate the results, it precluded a precise best response calculation. However, the effect on double peaks was ambiguous as the increase in water led to a decrease in double peak formation of the EOF marker but an increase concerning lumefantrine. This is supported theoretically as lumefantrine is very hydrophobic and insoluble in water, unlike thiourea, the EOF marker. Increasing the SDS concentration decreased the probability of double peaks and increased the retention time. The incomplete full factorial design, lacking axial points, limited the response optimiser to considering only low, high and central levels, preventing a continuous response surface fit. As a result, DoE5 was identified as the best option based on the lowest double peak probability and micelle retention time, despite having one of the longest overall retention times. Of course, as mentioned above, the DoE5 mobile phase had one of the longest retention times. However, this can still be considered as good, as in another study, the MEKC buffer had a separation time of 35 or 50 min, depending on whether the SDS had a concentration of 50 or 60 mmol/l, and the MEKC buffer had a water ratio of 90 % (v/v) (3). In comparison, the DoE5 mobile phase used here with 115 mmol/l SDS and 55 % (v/v) water could achieve a separation time of less than 20 min if there were no capillary problems (**data not shown; see also following data**).

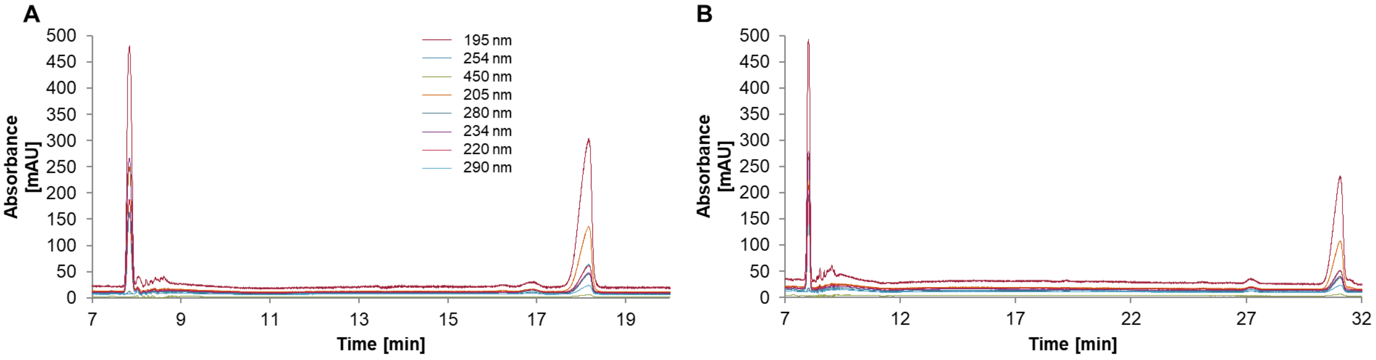

Supplement Figure 4 Part of the MEEKC optimisation results via DoE. Chromatograms show only the relevant part. For clarity, the legends are all the same as in **A)**. The capillary length was 43.1 cm (effective length: 33.1 cm). The sample was dissolved in mobile phase and loaded with 50 mbar for 6 s. The running condition was 20 kV for 35 min. In **A)** the MEEKC mobile phase consisted of 7.69 mmol/l NaH_2PO_4, 85 mmol/l SDS, 0.91 % (v/v) n-heptane, 23.74 % (v/v) butan-1-ol, 20.35 % (v/v) acetonitrile, 55 % (v/v) water, pH* 8.47. The sample consisted of 1.61 mg thiourea + 0.63 mg lumefantrine + 0.88 mg menaquinone 4 + 1 ml buffer. In **B)** the MEEKC mobile phase consisted of 7.69 mmol/l NaH_2PO_4, 115 mmol/l SDS, 0.91 % (v/v) n-heptane, 23.74 % (v/v) butan-1-ol, 20.35 % (v/v) acetonitrile, 55 % (v/v) water, pH* 8.49. The sample consisted of 2.51 mg thiourea + 0.63 mg lumefantrine + 1.00 mg

3.3. Investigations to improve repeatability of retention time

a. Retention shifts problems

For verification, the DoE5 mobile phase experiments were repeated with different set ups. However, retention time aberrations proved to be troublesome (**data not shown**). For example, comparison of the retention times of one sample, performed as the first three runs of each sequence on two days, showed a divergent retention time behaviour (**Supplement Figure 5**). To illustrate the effect on the retention factor, it was calculated and shown here for lumefantrine. For day 1, lumefantrine had an intraday retention factor of 16.12 with an SD of 0.73 and a CV of 4.56. For day 2, the intraday retention factor was 18.05 with an SD of 0.36 and a CV of 2.01. The inter-day retention factor was 17.08 with an SD of 1.1264 and a CV of 6.5942. As shown, this difference and CV are too high to be a reliable factor for hydrophobicity.

The used formula for the retention factor was under the simplified assumption that the retention time of the micelles is extremely long.

$$K = \frac{t_r - t_{eof}}{t_{eof} * \left(1 - \frac{t_r}{t_{MC}}\right)} \; or \; t_{MC} \Longrightarrow \infty \; K = \frac{t_r - t_{eof}}{t_{eof}}$$

$t_r = retention\ time\ of\ analyte$

$t_{\ eof} = Migration\ time\ of\ EOF\ marker \sim Flow\ rate\ (EOF)$

$t_{MC} = migration\ time\ of\ micelles$

A partial repeat after cleaning of the same capillary resulted in not baseline separated peaks (**data not shown**), and an additional cleaning improved partial the baseline separation, but with strong tailing (**data not shown**). To investigate the pH effect on the EOF, other mobile phases with the same composition but pH* 9.01 or 7.98 were prepared. However, both did not do well (**data not shown**). These new experiments could also show that diisodecyl phthalate is also a suitable micellar marker (**data not shown**). Interestingly, with a mobile phase at pH* 7.98, four runs could be performed on a freshly prepared capillary before the EOF strength disappeared (**data not shown**). This is contrary to theory, as a higher pH should increase the EOF strength and may indicate that there are other reasons behind this phenomenon.

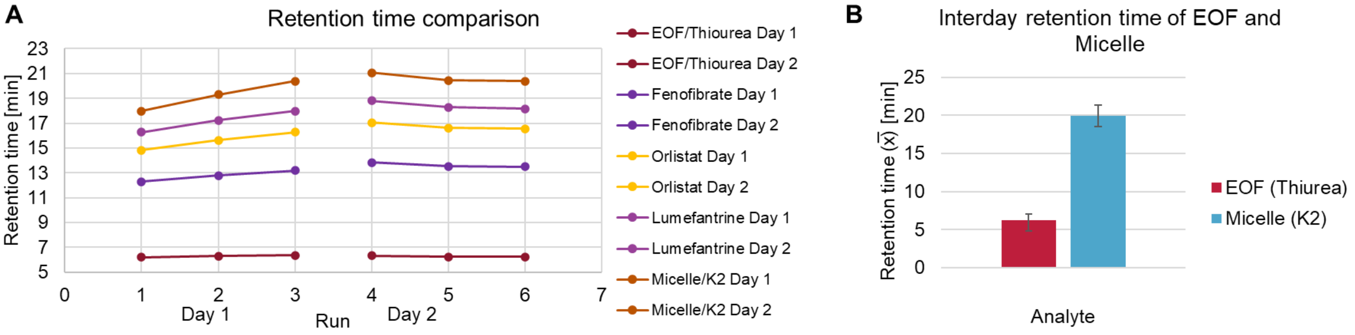

Supplement Figure 5 Retention time comparison of two identical samples with the same mobile phase on two different days. Both triplicate sample runs were performed as the first three runs of each sequence on the respective days. The mobile phase was the DoE5 mobile phase. **A)** shows the trend of the retention time of the analytes/markers. Due to the software, the run number on the x-axis is counted through. Run 4-6 on day 2 is actually run 1-3 on that day. **B)** Based on the two sequences with their EOF marker and their micellar marker menaquinone 4 (K2). Performed on two different days. Note that n = 6. The standard deviation is 0.0605 for the EOF and 1.0151 for the micelle.

b. Other actions

The main remaining problem was the lack of reproducibility of the retention times, as the retention factor depends on them. Methanol rinsing was tested to determine whether it could remove any assumed remaining impurities from the capillary wall, but it was not useful (**data not shown**). One other attempt was to increase the temperature during the conditioning step up to 50°C of the capillary chamber. This resulted in a partial recovery of the EOF strength, but also in an abnormal increase of the signal at the 195 nm wavelength (**data not shown**). Evaporation of higher amounts of the organic additives can be also dismissed as this was not a timed effect.

3.4. Conclusions

As mentioned in the main manuscript, three publications were found with the same aim of separating very hydrophobic compounds by ME(E)KC (4–6). One aimed to separate only the hydrophobic vitamins A, D, E and K and was successful using a MEEKC buffer consisting of 40 mmol/l borate buffer, 80 mmol/l SDS, 5 % (v/v) butan-1-ol, 0.8 % (v/v) octane, 15 % (v/v) propan-2-ol . This publication was found during the writing of the introduction to this study and has not yet been tested (6). Another publication was also found during the writing of the introduction, but dated already from 1999 (4). The mobile phase used consisted of 0.81 % (w/w) octane, 6.61 % (w/w) butan-1-ol, 3.31 % (w/w) SDS and 89.27 % (w/w) 10 mmol/l sodium tetraborate buffer at 40°C. This allowed the separation of a mixture of water-soluble vitamins and the non-water-soluble vitamins A and D (4). It should be noted that these two publications only aimed at separation and not at high reproducibility with stable retention times for the determination of the retention factor. Furthermore, as it was with the publications by Klotz *et al.* (2) and Yin *et al.* (7), it is not guaranteed that these newly found mobile phases are truly capable of separation, as there setups were also unable to separate the hydrophobic analytes used in this study without modification. A recent publication from 2022 (5) shows that the

separation of highly hydrophobic compounds such as menaquinone-7 (xlogP = 9.58; ALOGPS 2.1) and α-tocopherol (xlogP = 8.84; ALOGPS 2.1) can be achieved, and even preconcentration techniques can be implemented (5). However, it is a different approach by establishing a nanoemulsion (NE) MEEKC. For clarification, even though they are called microemulsions and nanoemulsions, a microemulsion is defined as having particle sizes of 20-50 nm and a nanoemulsion with particle sizes of 100-1000 nm. An NE is prepared by diluting an oil-in-water microemulsion, called “cold dilution”. These NEs require less surfactants and are thermodynamically unstable. Different NE compositions were also investigated, and it was found that, in terms of signal intensity, it can be useful to have one NE for the sample matrix and another for separation. Despite the announced reproducibility, no real data were shown. However for the two preconcentration variants investigated, linearity was demonstrated with n = 6 for each concentration point, so it can be assumed that the repeatability of the peak area is sufficient (5). However, the repeatability with respect to retention time is unknown, which is the important point/objective of this work at hand.

4. References

1. Hefnawy M, Alzamil A, Abuelizz H, AlShehri M. New bioanalytical microemulsion Electrokinetic chromatography method for the simultaneous determination of Trifluridine with its metabolites and Tipiracil in rat plasma: Application to pharmacokinetic studies. Saudi Pharm J 2019; 27(8):1075–84.

2. Klotz WL, Schure MR, Foley JP. Determination of octanol–water partition coefficients of pesticides by microemulsion electrokinetic chromatography. Journal of Chromatography A 2001; 930(1-2):145–54.

3. Ehala S, Vaher M, Kaljurand M. Separation of aromatic hydrophobic sulfonates by micellar electrokinetic chromatography. J Chromatogr A 2007; 1161(1-2):322–6.

4. Altria KD. Application of microemulsion electrokinetic chromatography to the analysis of a wide range of pharmaceuticals and excipients. Journal of Chromatography A 1999; 844(1-2):371–86.

5. Pieckowski M, Kowalski P, Olędzka I, Roszkowska A, Plenis A, Bączek T. Nanoemulsion supported microemulsion electrokinetic chromatography coupled with selected preconcentration techniques as an approach for analysis of highly hydrophobic compounds. J Chromatogr A 2022; 1677:463339.

6. Sánchez JM, Salvadó V. Comparison of micellar and microemulsion electrokinetic chromatography for the analysis of water- and fat-soluble vitamins. Journal of Chromatography A 2002; 950(1-2):241–7.

7. Yin C, Cao Y, Ding S, Wang Y. Rapid determination of water- and fat-soluble vitamins with microemulsion electrokinetic chromatography. J Chromatogr A 2008; 1193(1-2):172–7.
